# Risk of intracranial hemorrhage in patients using anticoagulant therapy for atrial fibrillation after cerebral microbleeds combined with acute ischemic stroke: a meta-analysis

**DOI:** 10.3389/fneur.2024.1372231

**Published:** 2024-03-15

**Authors:** Bingqing Zhao, Ye Yuan, Zheng Li, Ying Chen, Yali Gao, Baoling Yang, Jingyi Wu, Weihua Jia

**Affiliations:** ^1^Department of Neurology, Beijing Shijingshan Hospital, Shijingshan Teaching Hospital of Capital Medical University, Beijing, China; ^2^University of Glasgow, Glasgow, United Kingdom

**Keywords:** cerebral microbleeds, anticoagulation, intracerebral hemorrhage, recurrent stroke, systematic review

## Abstract

**Objective:**

To evaluate intracerebral hemorrhage (ICH) risk in patients with ischemic stroke (IS) and cerebral microbleeds (CMBs) undergoing anticoagulation therapy for non-valvular atrial fibrillation (AF).

**Methods:**

We conducted a comprehensive search across multiple databases, including Embase, PubMed, Cochrane, UpToDate, Scopus, WOS, and SinoMed. The search covered observational literature published from each database inception until February 1, 2023. We analyzed the prevalence of CMBs during the follow-up period, compared future ICH risk between patients with and without baseline CMBs (CMBs presence/absence, ≧5 CMBs), and examined factors influencing ICH occurrence in patients with CMBs. Also studied recurrent stroke during anticoagulation therapy, the risk of future ICH when white matter hyperintensity (WMH) and CMBs coexist, and the effects of anticoagulants vitamin K antagonists (VKAs) and direct oral anticoagulants (DOACs) on future ICH.

**Results:**

We included 7 articles involving 5,134 participants. The incidence of CMBs was 24%; baseline CMBs were associated with an increased ICH risk compared to patients without CMBs. ICH—risk was more significant in patients with baseline ≥5 CMBs. After anticoagulant therapy, ICH risk was higher than that of recurrent IS. The risk of future ICH was significantly increased with anticoagulant VKAs compared with NOAC.

**Conclusion:**

Anticoagulant therapy for ischemic stroke patients with non-valvular AF and CMBs increases future ICH risk. Discontinuing anticoagulation due to ICH risk should be avoided. NOACs are safe and effective for patients with CMBs and IS.

## 1 Introduction

Cerebral small vessel disease (CSVD) was mainly manifested on MRI as cerebral microbleeds (CMB), lacunar infarction (LI), white matter hyperintensities (WMHs), and enlarged perivascular space (EPVS) (1). CMBs are usually caused by CSVD (generally 2–5 mm, up to 10 mm diameter) or hemosiderin deposition in brain tissue. On imaging, CMBs manifest as round or oval scattered low signal shadows using gradient recalled echo (GRE) T2-magnetic resonance imaging (MRI) or susceptibility weighted imaging (SWI) (2, 3). A 9 years follow-up study showed that CMBs did not disappear with time (4), although prevalence increased with age. CMB incidence was 11% in people aged 60–69, 22% in ages 70–79, and 39% in ages 80 and older (5). Although most patients with CMBs do not exhibit clinical symptoms, they face an elevated risk of future neurological diseases such as stroke, cognitive impairment, and dementia (6, 7). Additionally, in cases underlying cardiovascular and cerebrovascular diseases, CMBs are positively correlated with an increased mortality risk (8). Thus, this condition requires significant attention in clinical practice.

Atrial fibrillation (AF) is a common arrhythmia that can increase the risk of ischemic stroke (IS). AF is also an independent risk factor for acute IS (AIS) (9). For AIS patients with non-valvular AF, anticoagulant therapy can effectively reduce the recurrence of IS; however, it increases the risk of related bleeding events (10).

In clinical practice, it is not uncommon for AIS patients with non-valvular AF to have CMBs simultaneously. A previous study reported new CMBs in 12.7% of patients 1 week after onset, and the presence of CMBs at baseline increased the risk of developing new CMBs (11). The main problems in managing these patients are advancing and retreating anticoagulant strategies and addressing bleeding symptoms. The presence of CMBs has been associated with a 1.5-fold increased risk of recurrent stroke [hazards ratio (HR), 1.5; 95% confidence interval (CI), 1.0–2.3], a four-fold increased risk of intracerebral hemorrhage (ICH) (HR, 4.2; 95% CI, 1.3–13.9), and a two-fold increased risk of all-cause death (HR, 2.1; 95% CI, 1.1–4.3) (12). Charidimou et al. (13) included 990 patients with IS using anticoagulants and with atrial fibrillation in a meta-analysis and found that CMBs at baseline increased the risk of symptomatic intracranial hemorrhage (OR = 4.16, 95% CI: 1.54–11.25) was not associated with recurrent IS (OR = 0.76, 95% CI: 0.40–1.45). Wilson et al. (14) highlighted that patients with AF, with or without CMBs, exhibited a significantly higher incidence of recurrent IS and vascular-related death than oral anticoagulant-related hemorrhagic stroke.

The above observations pose a dilemma for clinicians managing patients with CMBs and a significant indication for anticoagulant therapy. Balancing the risk of bleeding against the need for oral anticoagulant therapy (OAC) poses a significant clinical problem. Therefore, we conducted a comprehensive analysis of the published literature to investigate the future risks of ICH in IS patients with non-valvular AF who received anticoagulant therapy in combination with CMBs.

## 2 Methods

### 2.1 Protocol registration

We performed a systematic review and meta-analysis according to the Preferred Reporting Items for Systematic Reviews and Meta-Analyses guidelines. In addition, we registered the protocol of this systematic review with PROSPERO (CRD42023397029).

### 2.2 Data sources and searches

We conducted comprehensive searches in Embase, PubMed, Cochrane, UpToDate, Scopus, Web of Science, and SinoMed, regardless of language. The search strategy covered the period from database inception to February 1, 2023: “Cerebral Microbleed *” OR “CMB” OR “microbleed.mp” AND “ischeamic stroke” OR “stroke”/exp” OR “stroke” OR “stroke, acute ischemic” AND “anticoagulation” OR “anticoagulation*” OR “anticoagulant drugs” etc. Three reviewers (YY, YC, and YC) independently screened the titles and abstracts, and full texts were sourced for relevant articles. Inclusion criteria were assessed independently, and inconsistencies were resolved by consensus. The reference lists of included trials and other published meta-analyses were also reviewed for relevant articles.

### 2.3 Inclusion and exclusion criteria

Studies that met the following inclusion criteria were included: cohort studies with a prospective or retrospective design that provided a complete publication of the study and reported follow-up data on the development of future ICH and recurrent IS. The specific inclusion criteria were as follows: (1) adult subjects with recent non-valvular AF combined with AIS, usually requiring OAC to prevent stroke recurrence; (2) T2-GRE/SWI MRI indicating the presence of baseline CMBs; (3) quantification of the risk for each outcome concerning the presence of CMBs; (4) minimum follow-up duration of 1 year; (5) present risk assessment of symptomatic spontaneous ICH and IS during follow-up with valid definitions. (Risk of symptomatic spontaneous ICH (primary outcome) and IS during follow-up). The exclusion criteria were as follows: (1) where publication was a review, letters, editorials, or population-based studies; (2) unavailable outcome measures; (3) Duplicated study populations; (4) follow-up duration <12 months.

### 2.4 Data extraction

After our initial assessment for eligibility, BY and WJ independently completed a data extraction form. YY, a third reviewer, resolved any disagreements. We classified the studies as prospective or retrospective based on how data were collected. The extracted data included the following: first author, year of publication, country of origin, characteristics of participants such as age range, sex, prevalence of hypertension, number of participants and cases, duration of follow-up, MRI Sequence, imaging parameters, presence of CMBs, research method, and participants with intracranial hemorrhage events or participants with IS events during the follow-up period.

### 2.5 Risk of bias assessment

Two researchers (BZ and ZL) used the Newcastle–Ottawa scale (NOS) (15) to assess the quality of the included literature. The scale has three items: selection, comparability, and outcome, each consisting of several subentries, resulting in eight subentries for assessment. The maximum score was 9 points; a total score ≥7 was considered high-quality research.

### 2.6 Statistical analysis

Statistical analysis was performed using Review Manager 5.2 software. The pooled overall prevalence of CMBs was calculated based on the “metaprob” routine using exact binomial procedures. Our primary analysis quantified the strength of any association with outcomes of interest using odds ratios (OR) and 95% CI in patients with any CMB and ≧5 CMBs vs. no CMBs as the reference group. Given possible heterogeneity between studies, we pooled estimates from eligible studies using a fixed-effects model. Heterogeneity observed within and between studies was assessed using the Cochrane *Q* statistic, *p* ≤ 0.1, or *I*^2^ ≥ 50% for heterogeneity; *p* > 0.10, or *I*^2^ < 50% for the absence of heterogeneity. For the primary endpoint, sensitivity analyses were performed by removing one study per iteration to confirm that any single study did not influence the conclusions of this study. Egger’s test and funnel plot were used to detect the publication bias of the primary endpoint for the five or more included pieces of literature. *p* < 0.05 was considered statistically significant. The results of the meta-analysis were presented using forest plots.

## 3 Results

### 3.1 Study selection

**Figure 1:**
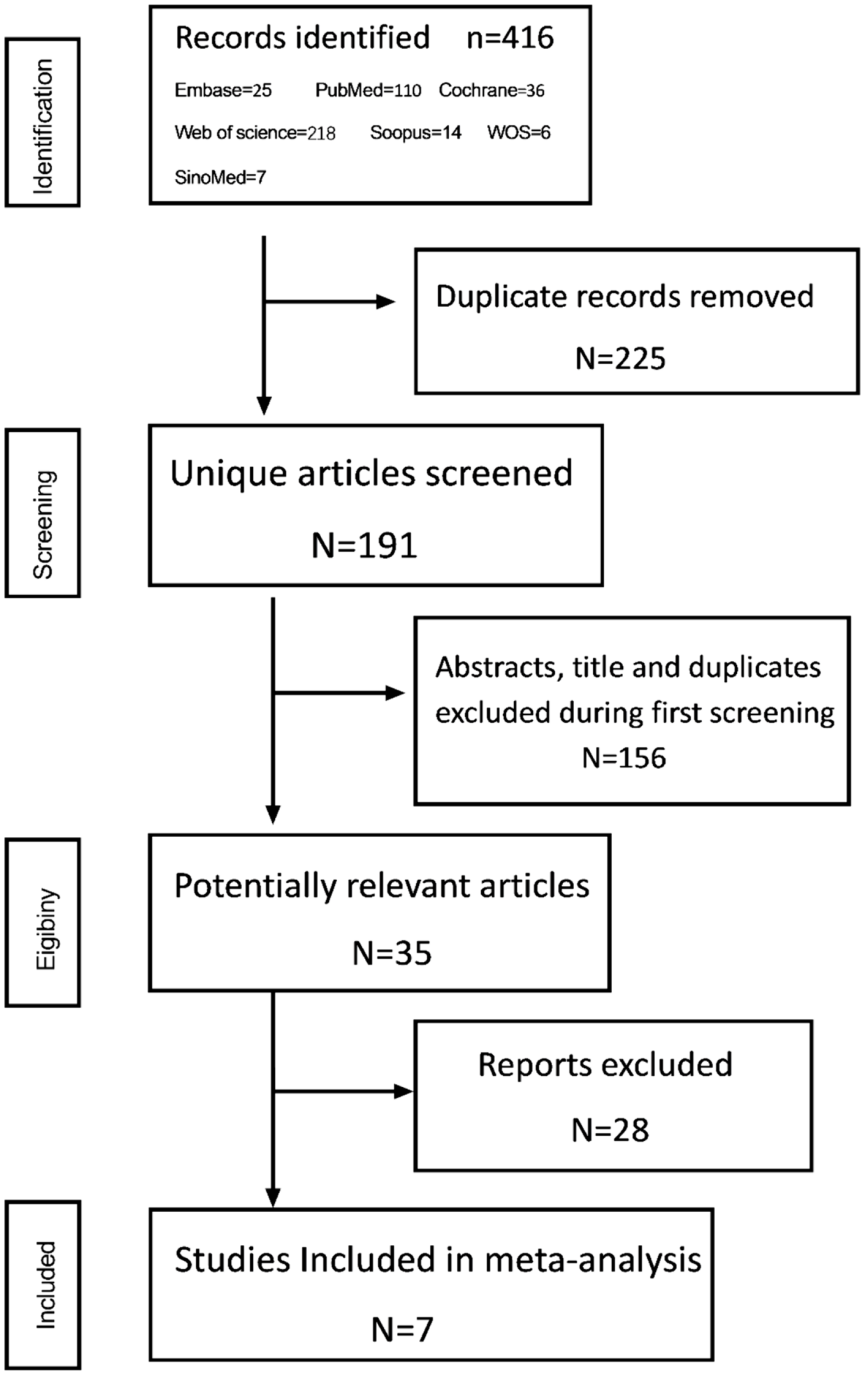
Flowchart of literature search and study selection.

A total of 426 articles were identified according to the retrieval formula, after excluding 225 duplicate articles and 156 for which the titles and abstracts did not meet the inclusion criteria. After a careful evaluation, 7 studies were finally included, and all were observational studies (Figure 1).

### 3.2 Study characteristics

A total of 7 articles (16–22), involving 5,134 participants, were included in our study. Table 1 summarizes the characteristics of the eligible studies. Among them, two from South Korea and one from China, Japan, Australia, Spain, and the United Kingdom. The median follow-up time ranged from 24 months (the follow-up period was 17–30 months). The participants included 2,902 male patients, 3,578 patients with a history of hypertension, and 1,251 patients with CMBs at baseline. All studies used T2-GRE or SWI MRI at baseline for CMB detection, with slightly different imaging parameters. Patients received oral anticoagulants of vitamin K antagonists (VKA) or direct oral anticoagulants (DOACs). Moreover, 6 of the 7 studies were prospective (16–21).

**Table 1:** Characteristics of included studies on prevalent cerebral microbleeds.

First author	Year of publication	Country	Sample size	Sex (% male)	Mean age (SD)years	Median follow-up time (month)	Hypertension (%)	Any cerebral microbleed	MRI Sequence	ICH events	IS events	Anticoagulant	Research method
Wagner (16)	2022	Australia	307	167	78.1 ± 9.2	24.4	236 (76%)	86	SWI	7	21	NOACs, VKAs	Prospective
Choi (17)	2020	Korea	1742	926	73 ± 9.7	16.4	1,048 (60%)	393	T2*	16	64	NOACs, VKAs	Prospective
Soo (18)	2019	China	237	140	742 ± 8.9	24	193 (81%)	84	SWI 3.0 T	4	12	VKAs	Prospective
Martí-Fàbregas (19)	2019	Spain	806	449	77.6 ± 6.5	24	694 (74%)	207	SWI 1/1.5/3.0 T	18	33	NOACs, VKAs	Prospective
Wilson (20)	2018	UK	1,436	859	76 ± 10	24	930 (64%)	300	T2 1.5/3.0 T	7	17	NOACs, VKAs	Prospective
Charidimou (21)	2016	Japan	102	73	76	17	85 (71%)	26	T2 1.5 T	3	14	NOACs, VKAs	Prospective
Song (22)	2014	Korea	504	288	70 ± 11	30	392 (77.8%)	155	T2 3.0 T	12	81	Warfarin	Retrospective

### 3.3 Quality assessment

NOS was used to assess the quality of the 7 studies (Table 2). Two papers scored 9 points (20, 22), four scored 8 points (17, 19), four scored 7 points (16), and two scored 6 points (18, 21). All seven articles are of high quality studies.

**Table 2:** Study quality assessment (Newcastle–Ottawa Scale).

	First author	Year of publication	Selection	Comparability	Exposure	Quality score
1	Wagner (16)	2022	***	**	**	7
2	Choi (17)	2020	***	**	***	8
3	Soo (18)	2019	**	**	**	6
4	Martí-Fàbregas (19)	2019	***	**	***	8
5	Wilson (20)	2018	****	**	***	9
6	Charidimou (21)	2016	**	**	**	6
7	Song (22)	2014	****	**	***	9

### 3.4 Meta-analysis results

The risk of intracranial hemorrhage after anticoagulation treatment of AIS combined with CMBs was investigated in 7 articles included in the current study. Including the incidence of CMBs (*n* = 7); risk of recurrent stroke in the future (*n* = 4); risk of ICH occurrence in the future of CMBs (*n* = 7), especially the risk of ICH occurrence in the future of CMBs ≧5 (*n* = 2); and the effect of anticoagulants on the risk of future ICH (*n* = 4).

#### 3.4.1 Incidence of CMBs and risk of future intracerebral hemorrhage

**Figure 2:**
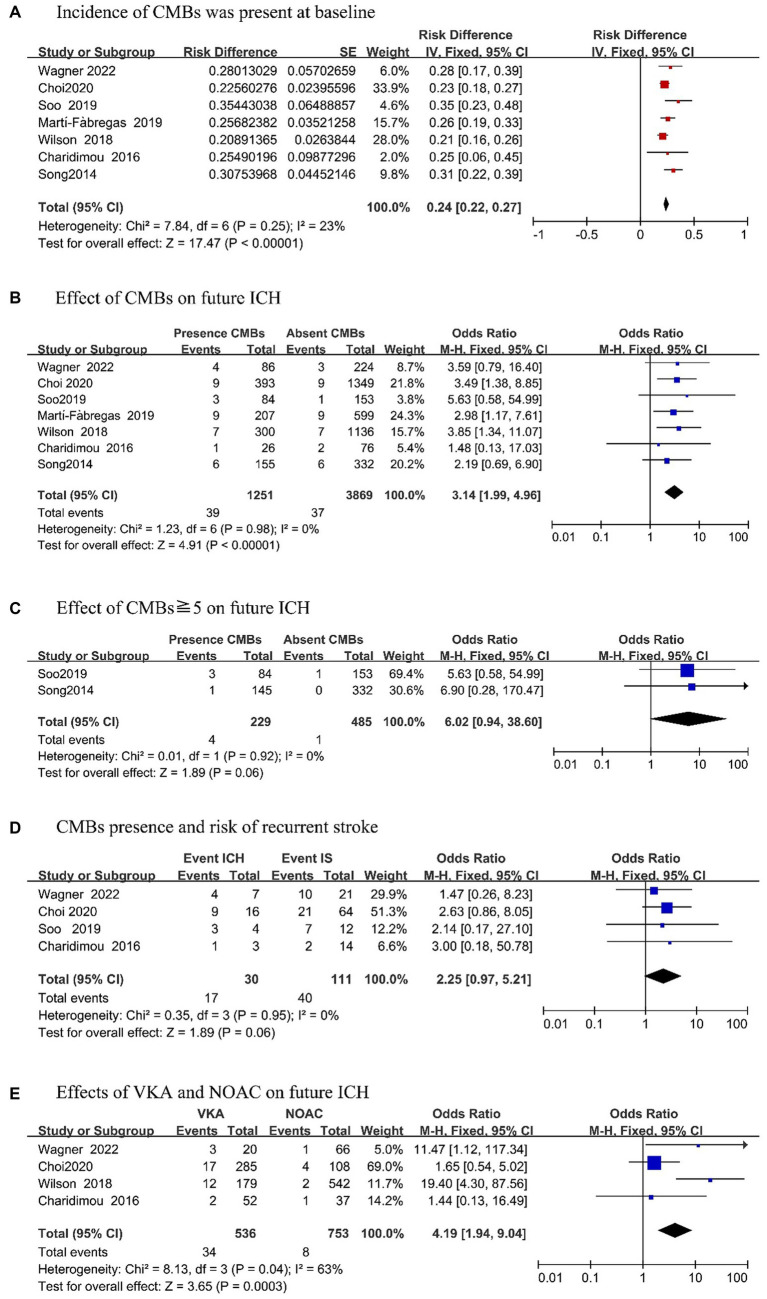
Outcomes of meta-analysis.

Among 7 included articles, the incidence rate of CMB was 24% (95% CI, 0.22–0.27; *p* = 0.25; Figure 2A). To more clearly analyze ICH risk in patients with CMBs at baseline, we reviewed 7 articles that provided detailed records of the presence of CMBs on MRI and the occurrence of ICH during follow-up (Figure 2B). Upon analysis, we found that patients with CMBs had an increased risk of future ICH as compared to patients without CMBs (OR, 3.14; 95% CI, 1.99–4.96; *p* = 0.98). Further analysis revealed that two studies (18, 22) mentioned the occurrence of ICH in patients with ≧5 CMBs (Figure 2C). Compared with patients without CMBs, the risk of ICH was more apparent in patients with CMB (OR, 6.02; 95% CI, 0.94–38.60; *p* = 0.92). There was no evidence of heterogeneity in this analysis.

#### 3.4.2 Risk of recurrent stroke

We analyzed the risk of future recurrent strokes after OAC in IS patients with CMBs (Figure 2D). Among the four studies (16–18, 21), it was found that during the follow-up period, the occurrence of ICH in the future was significantly higher than that of recurrent IS (OR, 2.25; 95% CI, 0.97–5.21; *p* = 0.95) in patients with CMBs at baseline.

#### 3.4.3 Effect of anticoagulants on the risk of future ICH

Finally, we investigated the future risk of anticoagulation in IS patients with CMBs (Figure 2E). We stratified the meta-analysis according to the type of OAC used, and four articles (16, 17, 20, 21) mentioned the effect of VKAs and DOACs in patients with CMBs. According to the combined effect size, in patients who took VKAs, the risk of ICH was significantly increased than that of NOAC patients (OR, 4.19; 95% CI, 1.94–9.04; *p* = 0.04) during the follow-up period.

## 4 Discussion

Our meta-analysis analyzed a specific population of AIS patients with non-valvular AF, who had CMBs at baseline as confirmed using MRI, and needed anticoagulant therapy to prevent future recurrent IS. Our study involved 7 articles in the Australia, Europe, and Asia. In nearly 5,000 IS patients with AF, the prevalence of CMBs at baseline was 24% (95% CI, 0.22–0.27; *p* = 0.25). The development of CMBs increases gradually over time, and most new CMBs develop gradually in patients with existing CMBs (11). However, new CMBs can also develop rapidly after AIS (23). As CMBs increase, the presence at baseline is more likely to develop into ICH while receiving OAC (24, 25). Our analysis of 7 studies revealed that CMBs at baseline in patients with AF receiving OAC had a significantly higher risk of future ICH (OR, 3.14; 95% CI, 1.99–4.96; *p* = 0.98). Charidimou et al. (26), among others, also reached similar conclusions as that in the current study. Their meta-analysis included nine clinical studies involving 1,552 patients followed for 6 months, demonstrating that those with CMBs at baseline had an increased risk of ICH (OR, 2.68; 95%CI, 1.19–6.01, *p* = 0.017). Compared to our study, this study showed a lower risk of future ICH.

To further analyze ICH risk, we stratified the burden of CMBs. After analyzing two studies involving CMBs ≥5, we found a significant increase in ICH risk with CMBs ≥5 (OR, 6.02; 95% CI, 0.94–38.60; *p* = 0.92). Choi et al. (17) also observed a correlation between the burden of CMBs and a high incidence of ICH, which can subsequently affect the risk of future cardiovascular events. Therefore, following up and monitoring disease progression is necessary for patients with a high burden of CMBs. However, due to the lack of data on the impact of CMB distribution on stroke risk, we could not perform the relevant analysis. Some scholars believe that lobar CMBs reflects cerebral amyloid angiopathy, and deep CMBs represent diseases caused by hypertension and arteriosclerosis (27, 28). Lobar CMBs may be more likely to develop ICH than deep CMBs (29). Especially in the case of OAC, the presence of lobar CMBs results in increased risk of ICH, while the risk of ICH was not found to be associated with presence of deep CMBs (26). Therefore, lobar CMBs can predict ICH risk (26, 30). However, some studies have found no significant correlation between the location of CMBs and ICH (31). Thus, more large-sample studies are needed in the future to determine the relationship between CMB burden, site distribution, and ICH. These studies will provide valuable insight into the best management of IS patients and CMBs requiring OAC.

Furthermore, compared with patients without CMBs, IS patients with CMBs had an increased risk of future ICH and recurrent IS (OR, 3.87; 95% CI, 0.91–16.4 and OR, 2.23; 95% CI, 1.29–3.85) (32). In a population-based study (33), 4,759 participants were followed up for an average of 4.9 years. CMBs at baseline accounted for 18.7% of participants (approximately 889 individuals) who exhibited CMBs overall. Of these participants, 72 had recurrent IS, and 11 had an ICH. This study suggested that the presence of CMBs at baseline was associated with an overall risk of recurrent stroke (HR, 1.93; 95% CI, 1.25–2.99), and the risk of stroke further increased with more CMBs. These studies indicate that CMBs, as a risk factor, increase the risk of future ICH and recurrent IS. Therefore, CMBs can be used as an independent predictor of recurrent stroke (HR, 3.66; 95% CI, 1.47–9.09; *p* = 0.005) (34).

The risk of ICH and recurrent IS may directly affect the anticoagulation strategy. Therefore, based on the four studies in this study, we analyzed that during the follow-up period, according to the analysis of recurrent stroke, 30 patients had ICH and 111 patients had recurrent ischemic stroke after AIS anticoagulant therapy. This indicates that the number of recurrent IS after anticoagulant therapy is higher than that of ICH. For patients with CMBs at baseline, 17 had ICH, 40 had recurrent IS, and the combined effect size showed that patients with CMBs at baseline had a higher incidence of long-term ICH than those with recurrent IS. Patients with CMBs at baseline and recurrent stroke during follow-up were analyzed and combined with the effect size, and it was found that the incidence of long-term ICH was higher in patients with CMBs at baseline (OR, 2.25; 95% CI, 0.97–5.21; *p* = 0.95). Similarly, in the NAVIGATE ESUS trial (11), 3,699 participants were treated with randomized, double-blind antithrombotic therapy. The *post hoc* analysis of the impact of CMBs showed that CMBs accounted for 11%, and the risk of stroke recurrence with CMBs at baseline increased by 1.5 times (HR, 1.5; 95% CI, 1.0–2.3), while the risk of cerebral hemorrhage increased by four times (HR, 4.2; 95% CI, 1.3–13.9). Therefore, patients with recurrent stroke appear to have an increased incidence of ICH compared with recurrent IS after anticoagulant therapy in CMBs patients at baseline. In the study by Charidimou et al. (26), involving 3,067 patients who experienced an IS/transient ischemic attack, the presence of CMBs at baseline increased the risk of recurrence of any stroke (OR, 2.25; 95% CI, 1.70–2.98). Moreover, subgroup analysis revealed that CMBs in the Asian population were more closely related to ICH (OR, 10.43; 95% CI, 4.59–23.72; *p* < 0.0001), while in the European and American populations, CMBs mainly increased the risk of IS recurrence (OR, 2.23; 95% CI, 1.29–3.85; *p* = 0.004). This suggests that ethnic differences may affect the predictive value of CMBs on recurrent stroke (35).

WMH, of presumed vascular origin, often coexists with CMBs in patients with IS and severe CMBs are often accompanied by more severe WMH (36, 37). At the same time, severe WMH is more common in patients with CMBs than those without CMBs (38). In recent years, both severe WMH and CMBs have been related to the occurrence and prognosis of ICH (39). Previous studies have shown that WMH can be an independent predictor of poor prognosis in patients with a hemorrhagic stroke, with increasing severity of WMH correlating with significantly higher mortality and disability rate at 90 days (40). A prospective study involving 153 patients from the Department of Neurosurgery and Neurology, West China Hospital, Sichuan University, China, observed over 5 years, revealed a significant increase in adverse long-term ICH outcomes when WMH and CMBs coexisted (OR, 2.124; 95% CI, 1.352–3.337; *p* = 0.002) (41). However, this study did not specifically address whether patients should use antithrombotic drugs. In a study by Martí-Fàbregas et al. (19) involving 121 patients, regarding antithrombotic therapy, CMBs and significant WMH coexisted, and this increased the risk of ICH reaching a rate of 3.76 per 100 patient-years. Unfortunately, due to insufficient data, our study was unable to analyze the long-term risk of ICH occurrence when WMH and CMBs co-existed and anticoagulants were used.

The prevention of recurrent stroke by OAC in patients with non-valvular AF has been widely recognized and recommended by guidelines for long. The higher relative bleeding risk of those anticoagulants is concerning. Notably, this study found that VKAs had an increased risk of long-term ICH as compared to DOACs (OR, 4.19; 95% CI, 1.94–9.04; *p* = 0.04). Similarly, a meta-analysis by Cheng et al. (42) found that the increased prevalence of CMBs was associated with warfarin use (OR, 1.64; 95% CI, 1.23–2.18) but not associated with DOACs (OR, 0.82; 95% CI, 0. 51–1. 33). Furthermore, Warfarin use has been associated with progression of CMBs burden (43), suggesting that patients with CMBs are at higher risk. Therefore, the benefits and risks should be evaluated when considering antithrombotic therapy for patients with CMBs at baseline on MRI (10). A small prospective study involving AF patients with CMBs at baseline who were treated with DOACs demonstrated that the duration of DOACs use was not significantly associated with the prevalence of CMBs and did not increase the risk of new CMBs (44, 45). Therefore, DOACs may have better efficacy and safety than warfarin (46). Current studies have not found a significant difference in the risk of ICH in IS patients with CMBs based on the type of OAC (DOACs or VKAs) (11).

Consequently, OAC should not be rejected solely because of the presence of CMBs (47). In cases of cerebral hemorrhage, there was some LI, and about 22.8% of the patients were discharged without any symptoms (48). However, whether the risk of bleeding is increased after anticoagulation in these patients needs further study. If the etiology of ICH has been identified and successfully treated, it is recommended to consider starting or restarting OAC therapy after 2–4 weeks, with DOACs being the preferred choice (49). Overall, DOAC drugs are increasingly used clinically and appear safe and effective for CMBs combined with IS. However, more large-sample, multi-center, high-quality randomized controlled trials are still needed to establish a more reliable basis for clinical application. The optimal stroke prevention strategy for patients with AF and IS should be elected accordingly.

This study has certain limitations. First, the incomplete or missing data on CMB distribution in the whole study makes it impossible to analyze the relationship between the location of CMBs and ICH; thus, it requires further study. Second, the types of OAC used during the follow-up period differed; most studies used warfarin, which limits the evaluation of DOACs efficacy. Third, due to insufficient data, our study lacks an examination of the coexistence of WMH and CMBs with recurrent IS. Therefore, future research should explore the relationship between the coexistence of WMH, CMBs, and recurrent stroke. Finally, future large-scale RCT studies are needed to evaluate the safety and efficacy of anticoagulant therapy in AIS patients with non-valvular AF concurrent CMBs.

A strength of this study lies in the use of high-quality data from prospective studies in most of the included literature (6/7), which were conducted with strict baseline evaluation and clinical follow-up. There was low heterogeneity between studies.

## 5 Conclusion

Our findings suggest that anticoagulated IS patients with non-valvular AF may have an increased risk of future ICH due to CMBs, especially with CMBs ≧5 or when WMH and CMBs coexist. However, anticoagulants cannot be discontinued because of the risk of ICH. DOACs appear to be safe and effective in patients with CMBs and IS.

## Author contributions

BZ: Conceptualization, Data curation, Formal analysis, Methodology, Software, Visualization, Writing – original draft, Writing – review & editing, Investigation, Validation. YY: Data curation, Formal analysis, Investigation, Methodology, Writing – review & editing. ZL: Data curation, Formal analysis, Investigation, Methodology, Writing – review & editing. YC: Data curation, Formal analysis, Investigation, Methodology, Writing – review & editing. YG: Data curation, Formal analysis, Investigation, Methodology, Writing – review & editing. BY: Data curation, Formal analysis, Investigation, Methodology, Writing – review & editing. JW: Formal analysis, Software, Supervision, Visualization, Writing – original draft, Writing – review & editing. WJ: Data curation, Formal analysis, Investigation, Methodology, Project administration, Supervision, Writing – review & editing, Conceptualization, Validation.
